# HnRNPA2B1 Aggravates Inflammation by Promoting M1 Macrophage Polarization

**DOI:** 10.3390/nu15071555

**Published:** 2023-03-23

**Authors:** Meiyao Meng, Yuxiang Cao, Yankang Zhang, Shuang Liu, Yinzhao Zhong, Dongmei Wang, Dali Li, Lingyan Xu, Xinran Ma

**Affiliations:** 1 Shanghai Key Laboratory of Regulatory Biology, Institute of Biomedical Sciences and School of Life Sciences, East China Normal University, Shanghai 200241, China; meiyao_meng@126.com (M.M.);; 2 Shanghai Frontiers Science Center of Genome Editing and Cell Therapy, Shanghai Key Laboratory of Regulatory Biology, School of Life Sciences, East China Normal University, Shanghai 200241, China; 3 Chongqing Key Laboratory of Precision Optics, Chongqing Institute of East China Normal University, Chongqing 401120, China; 4 Department of Endocrinology and Metabolism, Fengxian Central Hospital Affiliated to Southern Medical University, Shanghai 201499, China

**Keywords:** hnRNPA2B1, inflammation, macrophage polarization, colitis, obesity, mRNA stability

## Abstract

Macrophages have critical contributions to both acute and chronic inflammatory diseases, for example, bowel disease and obesity, respectively. However, little is known about the post-transcriptional regulatory mechanisms in macrophage-mediated inflammatory diseases. hnRNPA2B1 (A2B1) is an RNA binding protein for mRNA fate determination. We showed that hnRNPA2B1 mRNA levels were increased in colon in dextran sodium sulfate (DSS)-induced colitis mice and in epididymal white adipose tissue (eWAT) and spleen of high-fat-diet (HFD)-induced obese mice. Consistently, mice with haploinsufficiency of A2B1 (A2B1 HET) are protected against DSS-induced acute colitis and HFD-induced obesity, with decreased M1 macrophages polarization in colon, eWAT and spleen. Mechanistically, A2B1 mRNA and protein levels were increased in LPS-stimulated RAW 264.7 macrophages, and A2B1 enhanced RNA stability of pro-inflammatory genes *Tnfα*, *Il-6* and *Il-1β* for the regulation of macrophages polarization. Interestingly, A2B1 HET mice exhibited reduced white fat expansion, which was influenced by macrophages, since conditioned medium from macrophages with A2B1 manipulation significantly changed preadipocyte proliferation. Our data demonstrate that A2B1 plays a vital role in macrophage-mediated inflammation via regulating mRNA stability, suggesting that A2B1 may be served as a promising target for the intervention of acute and chronic inflammatory diseases.

## 1. Introduction

With the advancement of modern industry, the world’s population has become increasingly exposed to various environmental or artificial pollutants that cause complicated inflammatory responses, incurring an increased incidence of immune-related diseases and seriously endangering human health [1,2]. It has been well recognized that the onset and progression of immune-related diseases are associated with disturbed immune system function or imbalances in immune homeostasis [3,4]. Immune cells are actively involved in the regulation of immune homeostasis. Among immune cells, macrophages are important innate immune cells and antigen-presenting cells that initiate natural immune responses by recognizing risk factors in the microenvironment. Macrophages feature the unique capability of forming different polarization states in response to different cues to direct distinct immune responses [5]. Macrophages mainly have two subtypes of polarization, namely, classically activated (M1) and alternatively activated (M2); the former associates with inflammatory responses and produces multiple pro-inflammatory cytokines, whereas the later associates with anti-inflammatory cytokines involved in tissue repair. Of note, M1 and M2 macrophages are interconvertible upon different physiological and pathological stimuli, which have important contributions in the development of inflammatory diseases [6]. Thus, it is important to elucidate the regulatory mechanisms of macrophage polarization in inflammatory diseases.

As a large family of RNA-binding proteins, heterogeneous nuclear ribonucleo-proteins (hnRNPs) play important functions in mRNA fate determination, including variable splicing of precursor mRNA, mRNA polyadenylation, regulation of mRNA stability and nucleo-cytoplasmic transportation, and thus, they are actively involved in all aspects of physiological and pathological conditions, such as cancer, immunity and metabolism [7]. hnRNPA2B1 (A2B1), a member of the hnRNPABs subfamily, consists of two structural homologous proteins (hnRNPA2 and hnRNPB1) characterized by a tight sequence correlation and conserved domain structure, with B1 having 12 additional amino acids at N terminus compared with A2. A2B1 is an RNA-binding protein that affects the localization, shearing, stability, translation and other biochemical functions of RNA [8,9]. It has recently been reported that A2B1 plays an important role in antiviral innate immune responses by recognizing viral DNA, which initiates type I interferon production and promotes nucleus exportation of mRNAs of viral DNA sensors cGAMP synthase (cGAS) stimulator of interferon genes (STING) for amplification of antiviral immune responses [10]. We have previously shown the involvement of A2B1 in obesity and metabolic diseases by regulating mRNA stability of mitochondrial and thermogenic gene programs in inguinal fat (iWAT) [11,12,13]. As obesity features low-grade chronic inflammation, these previous studies indicated a critical role of A2B1 in immune homeostasis, during which process, macrophages are indispensable. However, it is not clear how A2B1 impacts macrophage-mediated inflammatory functions and their polarization in acute and chronic inflammatory diseases.

We firstly found that A2B1 transcriptional levels are altered in mice tissues under acute and chronic inflammatory disease models and LPS-treated macrophages. In addition, to further examine the relationship between A2B1 and inflammatory diseases, we constructed mice with haploinsufficiency of A2B1 (A2B1 HET mice) and assessed the influence of A2B1 reduction in both dextran sodium sulfate (DSS)-induced colitis and high-fat-diet (HFD)-induced obesity mice model using wild-type (WT) and A2B1 HET mice by analyzing macrophages in colon, epididymal white adipose tissues and spleen via flow cytometry. Mechanistically, we studied the changes in A2b1 levels in M1 macrophages and its effects on pro-inflammatory gene mRNA stability, as well as investigated the influence of macrophage polarization on white preadipocyte proliferation. Overall, we demonstrated that A2B1 is mainly associated with development of acute and chronic inflammatory diseases at least partially by regulating M1 macrophages.

## 2. Materials and Methods

### 2.1. Cell Lines and Cell Culture

RAW264.7 cells (ATCC) were incubated in DMEM medium (1% penicillin/streptomycin and 10% FBS) in a cell culture incubator containing 95% air and 5% CO_2_ at 37 °C.

### 2.2. Mice Experiments

Mice were housed under a 12 h light/dark cycle and guaranteed a living temperature of 22 °C. Mice were provided with adequate water and food. Genotyped mice (Hnrnpa2b1 heterozygous mice) used in this study were generated by Shanghai Bioray Laboratory.

Dextran sodium sulfate (DSS) (MP Biomedicals, Aurora, CO, USA) is applied to induce acute colitis. Briefly, mice were given 3% DSS (ultra-pure water configuration) for 7 days. Each group of mice was observed and weighed daily. For obesity model, mice at 8 weeks old were fed with high-fat diet (HFD) (D12492, Research Diet, New Brunswick, NJ, USA) or normal chow diet (NCD) for 10 weeks.

### 2.3. Disease Activity Index (DAI) Scores Parameters

The DAI was calculated from the total score of three aspects (body weight loss, stool consistency, gross bleeding) as previously described [14].

### 2.4. RNA Extraction, Quantitative PCR

The cultured cells or mouse tissues were first lysed with RNAiso Plus (TaKaRa, Osaka, Japan) or homogenized and then lysed. The nucleic acid was extracted with chloroform, and precipitated with isopropanol. After reverse transcription Prime-Script^TM^ RT Master Mix (TaKaRa, Osaka, Japan), real-time fluorescence quantitative detection was performed with Roche LightCycler480 (Roche, Basel, Switzerland). Data normalized to β-actin level, using 2 ΔΔ. Computation of relative expression of RNA by CT method. Table S1 listed the primers used in the real time PCR. The experiment was repeated three times.

### 2.5. Western Blotting Analysis

After the sacrifice of mice, the tissue was weighed, and the RIPA buffer was added at the ratio of 1:20. The RIPA buffer was RIPA lysate (P0013B, Beyotime Biotech Biotech, Shanghai, China), protease inhibitor, phosphatase inhibitor, PMSF and DTT. After the beads are added, the tissue homogenate is centrifuged for three times and the supernatant is sucked for boiling the protein. All protein concentration of the samples was measured using the NanoDrop (Nano Dorp one, Thermo Scientific, Waltham, MA, USA). Protein samples were added and run-on SDS-PAGE and transferred to a NC membrane (66485, Pall, Port Washington, NY, USA) and then incubated with hnRNPA2B1 (ab31625, Abcam, Cambridge, MA, USA), Tubulin (AF5012, Beyotime Biotech Biotech, Shanghai, China), Phospho-NF-κB p65 (CST#3033, Cell Signaling Technologies, Boston, MA, USA) or NF-κB p65 (CST#8242, Cell Signaling Technologies, Boston, MA, USA) antibodies.

### 2.6. Cell Isolation and FACS Analysis

Regarding the stromal vascular component (SVF) isolation from adipose tissues, tissues were cut with scissors and digested with collagenase (14065056, Gibco, New York, NY, USA) in HBSS (14065056, Gibco, New York, NY, USA) in a 37 °C shaking water bath. Cells from spleen were obtained by direct grinding spleen tissue without enzymatic digestion and passed through cell filter and centrifuged before resuspension in red cell lysis buffer prior to further analysis.

For FACS analysis, after red blood cell lysis, Cells were collected at 4 °C and then resuspended in block buffer with 0.2%BSA in PBS and Anti-mouse CD16/32 (TruStain FcX, Biolegend, San Diego, CA, USA) for 0.5 h. Subsequently, cells were stained with surface antibodies at 4 °C for 30 min. Following antibodies were all purchased from Biolegend: PE anti-mouse F4/80, PE/Cyanine7 anti-mouse CD80, Alexa Fluor anti-mouse CD206, APC anti-mouse CD45, PE anti-PPmouse CD140a (PDGF Receptor a). Immune cells were firstly isolated using mouse CD45+ nanobeads (Biolegend, San Diego, CA, USA). Flow cytometry data were collected using Fortessa (BD LSR Fortessa, Franklin Lakes, NJ, USA). FACS analysis were carried out using FlowJo-V10 (FlowJo LLC, Ashland, OR, USA).

### 2.7. Insulin and Glucose Tolerance Test (ITT and GTT)

GTT was performed after mice were fasted overnight. Mice were intraperitoneally injected with glucose solution (Sigma, St Louis, MI, USA, 1.25 g/kg body weight) and blood glucose levels were measured at indicated time points. For ITT experiment, insulin (Sigma, St Louis, MO, USA, 1.25 U/kg body weights) was given by intraperitoneal injection and blood glucose levels were measured at indicated time points after the injection.

### 2.8. Serum Parameters Analysis

Serum was analyzed with the assay kit for total triglyceride, total cholesterol, HDL-C, LDL-C or non-esterified fatty acids purchased from Nanjing Jiancheng Bioengineering Institute.

### 2.9. Histological Analysis and Histopathological Score

Tissue was fixed overnight in neutral buffer formalin (HT501128, Sigma, St Louis, MI, USA), paraffin embedded, 5 μm thick and stained with hematoxylin and eosin (C0105S, Beyotime Biotech, Shanghai, China). The scale bars for the images shown in figure represents 200 μm. Immunohistochemistry was performed using standard protocols on deparaffinized sections using anti-F4/80 antibody (gb11027, Servicebio, Wuhan, China). Photographs were taken using an optical microscope (Ci-L, Nikon, Tokyo, Japan). Adipocyte sizes were analyzed by Image J. Briefly, at 100× magnification, five random fields of each slide were captured. Immunohistochemical analysis was performed using ImageJ (National Institutes of Health, Bethesda, ML, USA).

The histochemical score of colitis was obtained by summing the following two parts: (a) Epithelial structure score: normal = 0, cup shape cell loss = 1, goblet cell loss = 2, crypt loss = 3, crypt mass loss = 4; (b) Immune cell infiltration score: no infiltration = 0, infiltration around the crypt = 1, mucosal muscular infiltration = 2, mucosal muscular infiltration with mucosal thickening and massive edema = 3, submucosal infiltration = 4.

### 2.10. mRNA Stability Assay

RAW 264.7 macrophages were infected with adenovirus to mediate Hnrnpa2b1 overexpression or transfected with siRNA to knockdown Hnrnpa2b1 and treated with 0.1 μg/mL LPS (*E. coli* 0127:B8, Sigma, St Louis, MI, USA) for 4 h and then treated with actinomycin D (5 μg/mL, #A1410, Sigma, St Louis, MI, USA) for another 4 h. Cells were collected to prepare total mRNA, and RT-qPCR was performed as described above.

### 2.11. CCK-8 Assay

Enhanced CCK-8 assay (C0042, Beyotime Biotech, Shanghai, China) for cell viability were performed. Stromal vascular fractions (SVF) from eWAT were treated with culture medium from macrophages for 24 h. The microplate reader (Synergy NEO2, Bio-Tek, Winooski, VT, USA) was used to test the absorption value at 450 nm.

### 2.12. EdU Staining

The assay was performed using an EdU kit (C0071, Beyotime Biotech, Shanghai, China). SVF from eWAT were cultured in conditioned medium of Raw264.7 macrophages. Then, cells were incubated with EdU and permeated with strong permeability solution (P0097, Beyotime Biotech, Shanghai, China) for ten minutes. The cells were placed in the reaction mixture and incubated in the dark for 0.5 h and staining with Hoechst 33342 (C1022, Beyotime Biotech, Shanghai, China). The scale bars for the images shown in figure represents 100 μm. All images were acquired using an optical microscope (TS2FL, Nikon, Tokyo, Japan). Cell numbers were calculated using automated particle analysis in ImageJ (National Institutes of Health, Bethesda, ML, USA).

### 2.13. Co-Culture Analysis

Raw 264.7 macrophages were seeded in plates and treated with LPS for 4 h. The culture medium was then replaced with fresh DMEM cell culture medium without LPS for another 24 h, and the supernatant was added into eWAT SVF for further analysis.

### 2.14. Statistical Analysis

Statistics were computed with GraphPad Prism 8 software (GraphPad Software, La Jolla, CA, USA). Statistical differences between two groups were analyzed by Student’s *t*-test for unpaired data. Results are shown as mean ± SEM; ns, not significant; * *p* < 0.05, ** *p* < 0.01.

## 3. Results

### 3.1. Phenotypic Analysis of hnRNPA2B1 Heterozygous Mice

**Figure 1:**
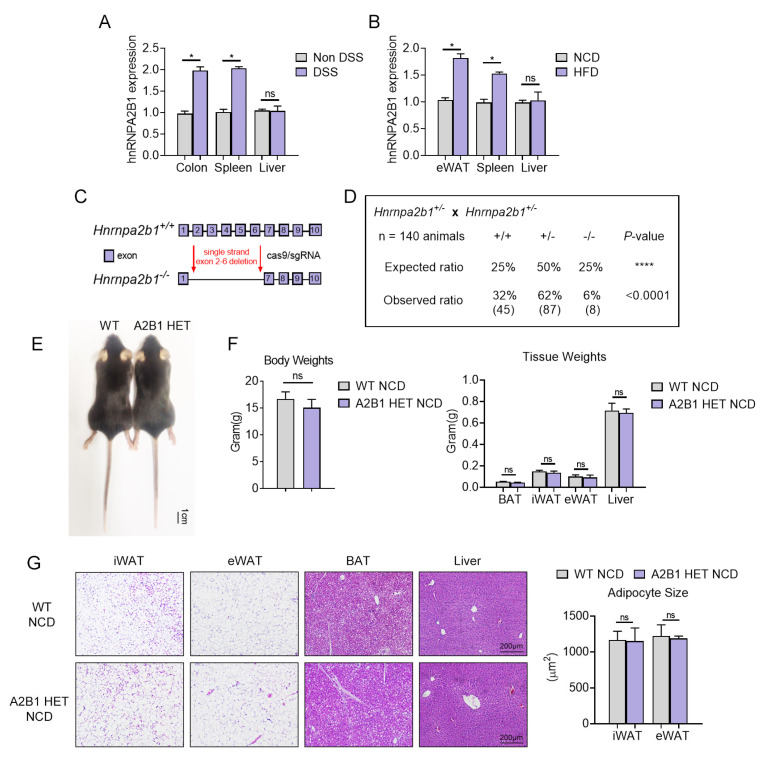
Phenotypic analysis of hnRNPA2B1 heterozygous mice. (**A**,**B**) Expressing profile of hnRNPA2B1 in different tissues of DSS-induced colitis mice and high-fat-diet (HFD)-induced obesity mice. (**C**) Schematic diagram for establishment of hnRNPA2B1 gene by CRISPR/Cas9 gene editing technology. (**D**) Birth rate of the different hnRNPA2B1 genotypes mice in NCD (normal chow diet). (**E**) Appearance of A2B1 HET NCD mice compared with that of WT NCD mice. (**F**) Body weights and weights of metabolic tissues including brown fat, inguinal fat, epididymal fat and liver. (**G**) Hematoxylin and eosin (H&E) staining of liver and adipose tissues. Results are shown as mean ± SEM; ns, not significant; * *p* < 0.05, **** *p* < 0.0001.

A2B1 has been shown to play crucial roles in immune response to viral DNA infection as well as in obesity [10,11]. As these pathological processes feature either acute or chronic inflammatory reaction, we are intrigued to examined the role of A2b1 in inflammatory diseases. We first established mice models of acute (DSS-induced acute colitis) or chronic (HFD-induced obesity) inflammatory responses and examined A2b1 levels in major organs. Interestingly, A2b1 mRNA levels were increased in colon and spleen of mice under acute colitis, and in epididymal fat and spleen of obese mice under HFD; however, liver tissue did not show any changes between groups (Figure 1A,B).

In order to better understand the effect of A2B1 on inflammation from a genetic perspective, we constructed hnRNPA2B1 genetic knockout mice by deleting exon 2–6 (Figure 1C) and found that the birth rate of A2B1 KO mice was very low and significantly below Mendel’s genetic law (Figure 1D), suggesting possible developmental defects in homozygous KO mice. In comparison, heterozygous mice (A2B1 HET mice), which were characterized by a similar physiological reduction in A2B1 levels that mimics decreased A2B1 levels in obesity [11], appeared normal and were indistinguishable from their wild-type littermates (Figure 1E). Detailed analysis revealed that A2B1 HET mice and WT mice did not differ in body weights and weights of inguinal fat (iWAT), epididymal fat (eWAT), brown fat (BAT) and liver (Figure 1F). Though it has been reported that the size of the fat cells in the iWAT of A2B1 HET mouse increased, and the insulin sensitivity was damaged at the age of 4 weeks [11], hematoxylin and eosin (H&E) staining revealed similar adipocyte sizes and liver morphology between WT and A2B1 HET mice at 8 weeks of age (Figure 1G), suggesting that whole-body A2B1 reduction may have minimal impact on fat upon adulthood.

### 3.2. hnRNPA2B1 Heterozygous Mice Were Protected from DSS-Induced Acute Colitis

**Figure 2:**
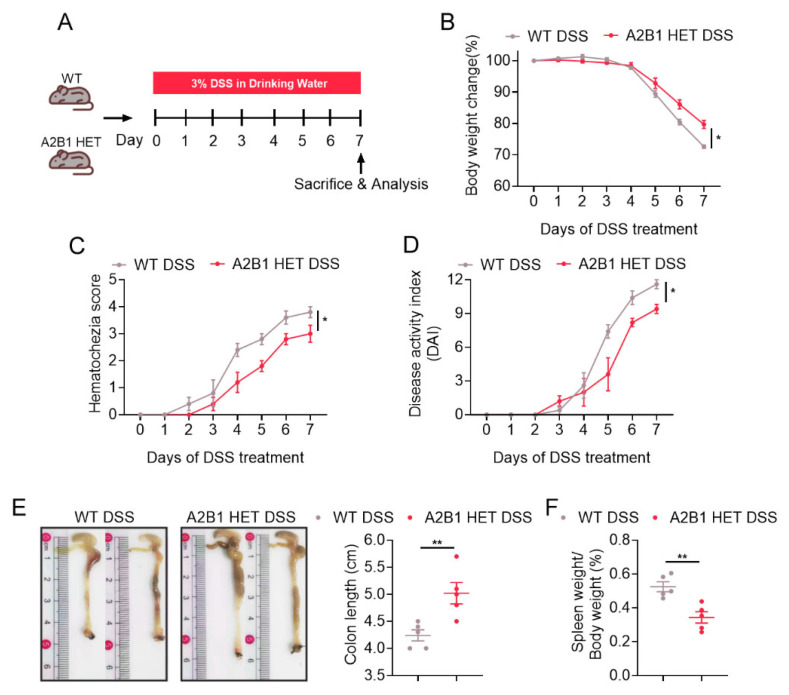
hnRNPA2B1 deficiency protects mice from DSS-induced acute colitis. (**A**) Schematic representation of the experimental design and timeline. (**B**) Percentage of body weight change from baseline after 3% DSS administration. (**C**) Hematochezia scores of DSS-treated WT and A2B1 HET mice. (**D**) Disease activity index (DAI) of DSS-treated WT and A2B1 HET mice. (**E**,**F**) Colon length (**E**) and spleen-to-body-weight ratio (**F**) of DSS-treated WT and A2B1 HET mice seven days post IBD induction. Results are shown as mean ± SEM; * *p* < 0.05, ** *p* < 0.01.

We thus further investigated the role of A2B1 in inflammatory diseases using WT and A2B1 HET mice of 8 weeks of age. Inflammatory bowel disease (IBD) is the characterization of Crohn’s disease and ulcerative colitis. The abnormal inflammation is manifested by increased intestinal inflammation, intestinal epithelial barrier damage and microbial imbalance [2]. The mucosal damage and increased gut permeability caused by systemic dysregulation of immune system are common features of IBD [15]. It has been reported that the activity of A2B1 was strongly associated with the occurrence and pathogenesis of IBD, and the pro-inflammatory-related factors are highly expressed in IBD patients [16]. In order to gain more insight into a possible role of A2B1 in colitis, A2B1 HET and WT mice were subjected to ad libitum water with 3% dextran sodium sulfate (DSS), a chemical disruptor of intestinal barrier homeostasis [17], to establish an acute colitis model, which is considered highly analogue to human IBD (Figure 2A). We found that along the pathological development of acute colitis, the weights of A2B1 HET DSS mice decreased more slowly compared to that of WT DSS mice (Figure 2B), indicating that A2B1 defect could ameliorate weight loss in mice, a parameter tightly associated with the severity of colitis. In addition, we observed that the activity of the WT DSS group began to decrease, and damp and cold body hair, loose stools, macroscopic bloody stools, and damp and cold oozing blood around the anus were shown on the fourth day of modeling, while A2B1 HET DSS group exhibited improved pathological conditions as evaluated by decreased hematochezia score and Disease Activity Index (DAI) score (Figure 2C,D). In addition, after 7 days into DSS induction, A2B1 HET DSS mice exhibited significantly longer colon length and lower spleen weights (Figure 2E,F). Overall, these results showed that hnRNPA2B1 haplo-insufficiency protected mice from DSS-induced colitis.

### 3.3. hnRNPA2B1 Heterozygous Mice Had Attenuated Colon Damage and Inflammation

**Figure 3:**
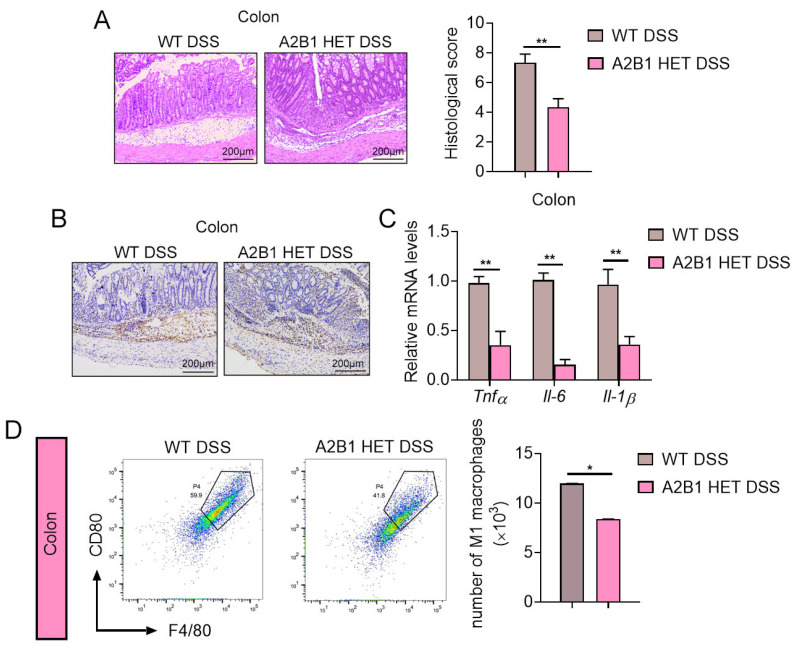
hnRNPA2B1 reduction attenuated colitis and inflammatory signs in DSS-induced IBD mice. (**A**) Colonic tissue H&E staining for the assessment of histological changes. (**B**) Immunohistochemistry analysis of F4/80 levels of colon tissues. (**C**) Relative proinflammatory genes mRNA levels in colon tissues of WT and A2B1 HET mice treated with DSS. (**D**) Flow cytometry and numbers of CD45+F4/80+CD80+ macrophages in colon of WT and A2B1 HET mice treated with DSS. Results are shown as mean ± SEM; * *p* < 0.05, ** *p* < 0.01.

We further performed histopathological analysis on colonic tissues of these IBD mice. A2B1 heterozygous DSS group showed fewer pathological changes in colon tissue, as characterized by less mucosal damage, increased goblet cells and crypts and significantly lower histological score as compared to wild-type DSS group (Figure 3A). F4/80 immunohistochemical staining of colon tissue suggested similar macrophages infiltration in colon of WT and A2B1 HET DSS mice (Figure 3B). We further examine M1 macrophages since it has been shown that dramatic increases in M1-polarized macrophages and pro-inflammatory genes are involved in the initiation of colitis [18]. Indeed, we found that the expression of pro-inflammatory genes was suppressed in the colon of DSS-treated A2B1 HET mice (Figure 3C). Furthermore, we used CD45 antibody-labeled nanospheres to derive immune cells in colon and then detected macrophage populations by flow cytometry analysis (FACS), which revealed decreased numbers of CD80-positive (M1-macrophage marker) macrophages in colons of A2B1 HET group (Figure 3D). These data suggested M1 macrophage polarization was decreased in A2B1 HET mice upon colonic immune insult, which may underlie the ameliorated pro-inflammatory responses and colon homeostasis in HET mice in DSS-induced colitis.

### 3.4. hnRNPA2B1 Heterozygous Mice Were Protected from HFD-Induced Obesity and Hyperlipidemia

**Figure 4:**
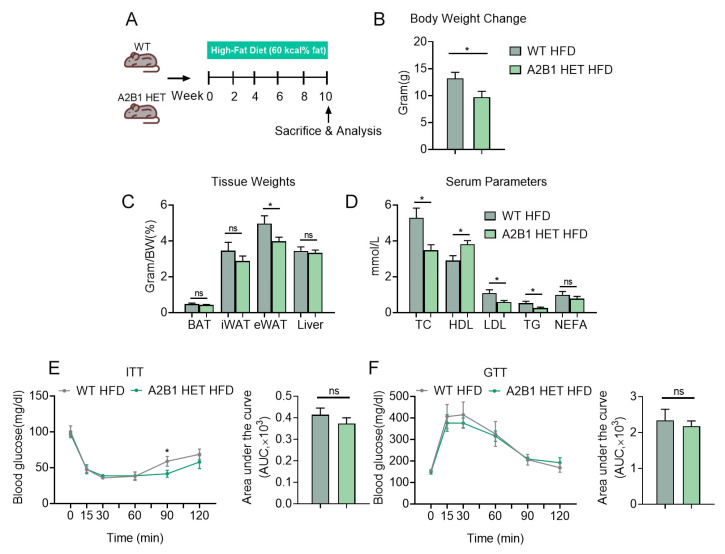
hnRNPA2B1 heterozygous mice under high-fat-diet-induced obesity. (**A**) Schematic representation of the experimental timeline and design. (**B**–**E**) Body weight change (**B**), weights of metabolic tissues including brown fat, inguinal fat, epididymal fat and liver (**C**), serum parameters including TC, HDL, LDL, TC and NEFA levels (**D**), ITT (**E**) and GTT (**F**) of WT and A2B1 HET mice after high-fat diet for 10 weeks. Results are shown as mean ± SEM; ns, not significant; * *p* < 0.05.

We next investigated the role of A2B1 in chronic inflammation by subjecting WT and A2B1 HET mice to HFD feeding to induce obesity and metabolic dysfunctions, which represents a state of chronic inflammation (Figure 4A). While the body weight of mice steadily increased during the 10-week HFD treatment as expected, we observed a marginal lower accretion in body weight change, with significantly reduced eWAT in A2B1 HET HFD mice (Figure 4B,C). Compared to WT, we found improved serum lipid parameters in A2B1 HET HFD mice, including reduced total cholesterol (TC), triglyceride (TG) and low-density lipoprotein (LDL) levels, as well as increased high-density lipoprotein (HDL) levels (Figure 4D), while their insulin sensitivity were unaltered as indicated by similar performances in insulin tolerance tests (ITT) and glucose tolerance tests (GTT) (Figure 4E,F). These data suggest that A2B1 reduction protected mice from obesity and hyperlipidemia induced by HFD.

### 3.5. hnRNPA2B1 Heterozygous Mice Were Resistant to Diet-Induced Inflammation and M1 Macrophage Polarization

**Figure 5:**
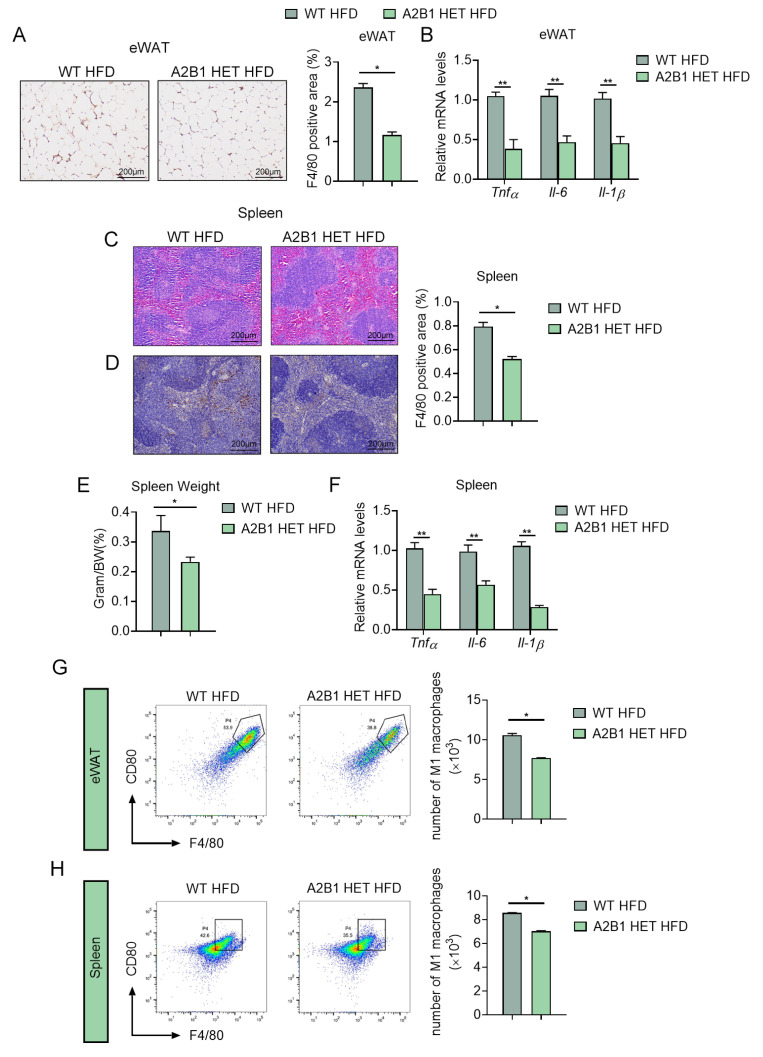
hnRNPA2B1 deficiency reduces M1 macrophages and inhibits inflammatory responses after HFD feeding. (**A**) Immunohistochemistry analysis of F4/80 levels in epididymal white adipose tissues. (**B**) Relative Tnfα, Il-6 and Il-1β mRNA expression levels in epididymal white adipose tissues of HFD-fed WT and A2B1 HET mice. (**C**–**F**) H&E staining of spleen (**C**), Immunohistochemistry analysis of F4/80 levels in spleen (**D**), Spleen-to-body-weight ratio (**E**) and Relative proinflammatory genes mRNA levels (**F**) in spleen of HFD-fed WT and A2B1 HET mice. (**G**,**H**) Representative images and numbers of CD45+F4/80+CD80+ macrophages in epididymal white adipose tissues (**G**) and spleen (**H**) of HFD-fed WT and A2B1 HET mice analyzed by FACS. Results are shown as mean ± SEM; * *p* < 0.05, ** *p* < 0.01.

Importantly, we found that the typical low-grade inflammation seen in fat tissues in obesity was suppressed in A2B1 HET HFD mice, as evident by reduced expression levels of F4/80 staining, as well as decreased inflammatory genes in eWAT of A2B1 HET HFD mice compared to controls (Figure 5A,B). The spleen weights of A2B1 HET HFD mice were also reduced (Figure 5E), accompanied with reduced apoptotic bodies and inflammatory cell infiltration under pathological analysis and decreased pro-inflammatory cytokine gene expressions via qPCR analysis in their spleen compared to WT HFD mice (Figure 5C,D,F). Furthermore, we obtained immune cells from eWAT and spleens using CD45 antibody-labeled nanoparticles and then examined macrophages in HFD-fed mice. We analyzed macrophages in WT and A2B1 HET HFD mice by FACS and revealed that the numbers of M1 macrophages were decreased in these tissues of A2B1 HET HFD mice (Figure 5G,H). These data suggest that A2B1 heterozygous mice were resistant to chronic inflammation and M1 macrophage polarization in obesity.

### 3.6. Modulation of hnRNPA2B1 in Macrophages Affects Preadipocyte Proliferation

**Figure 6:**
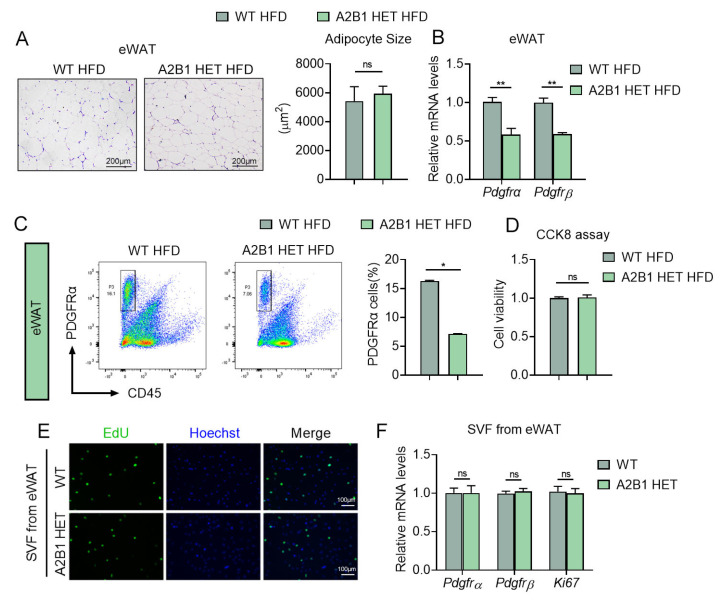
PDGFRα+ progenitor cell numbers are reduced in hnRNPA2B1 heterozygous mice. (**A**,**B**) H&E staining (**A**), Pdgfrα and Pdgfrβ mRNA levels (**B**) in epididymal white adipose tissues of HFD-fed WT and A2B1 HET mice. (**C**) Cell sorting of CD45-PDGFRα+ cells from eWAT SVF of HFD-fed WT and A2B1 HET mice by FACS. (**D**–**F**) Proliferation of SVF cells from eWAT of WT and A2B1 HET mice was evaluated by CCK-8 analysis (**D**), EdU analysis (**E**) and relative Pdgfrα, Pdgfrβ and Ki67 mRNA expression levels (**F**). Results are shown as mean ± SEM; ns, not significant; * *p* < 0.05, ** *p* < 0.01.

Interestingly, although A2B1 HET HFD mice were characterized by a reduced eWAT depot (Figure 4C), a closer look at the eWAT revealed that the adipocyte sizes were similar between A2B1 HET and WT HFD mice (Figure 6A), suggesting the reduced eWAT weights may be due to decreased preadipocytes hyperplasia, instead of mature adipocyte hypertrophy. Indeed, the qPCR and FACS analyses both showed decreased adipose progenitor marker *Pdgfrα* and *Pdgfrβ* in eWAT of A2B1 HET mice (Figure 6B,C). Importantly, preadipocytes from eWAT of A2B1 HET mice had similar cell proliferation rate, cell viability and preadipocyte markers compared to WT (Figure 6D–F), indicating the reduced preadipocyte hyperplasia of A2B1 HET eWAT may be due to extracellular signals, i.e., intercellular crosstalk.

**Figure 7:**
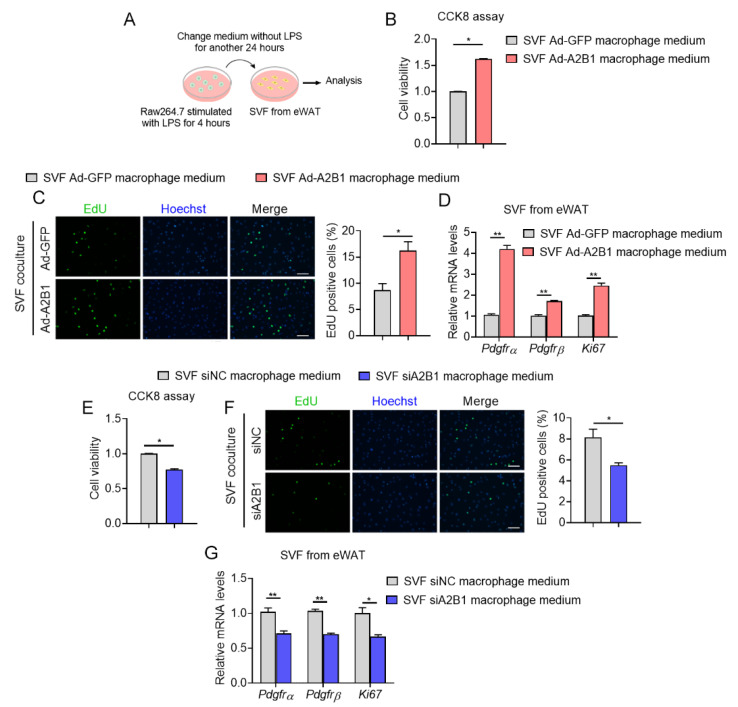
hnRNPA2B1 from macrophages promote preadipocyte proliferation. (**A**) Schematic illustration of the experiment design and workflow to evaluate proliferative capacity of SVF cells from eWAT treated with culture medium from LPS-treated Raw264.7 macrophages with A2B1 overexpression or knockdown. (**B**–**G**) Proliferative capacity of SVF cells from eWAT was analyzed by CCK8 (**B**,**E**), EdU assay (scale bar represents 100 μm) (**C**,**F**) and relative Pdgfrα, Pdgfrβ and Ki67 mRNA expression levels (**D**,**G**). Results are shown as mean ± SEM; * *p* < 0.05, ** *p* < 0.01.

It has been reported that proinflammatory cytokines such as TNFα, PEG2 and IL17, promote cellular proliferation in various cell types [19,20,21]. Since we have found that A2B1 altered levels of proinflammatory genes in macrophages, we thus examined whether modulation of A2B1 in macrophages may crosstalk with preadipocytes and affect their proliferation. We achieved A2B1 overexpression (Ad-A2B1 or Ad-GFP) or A2B1 knockdown (siA2B1 or siNC) in LPS-stimulated RAW264.7 macrophages. The conditioned culture mediums from all groups were collected and added into SVF from eWAT for further analysis (Figure 7A). Of note, culture medium from A2B1-overexpressed macrophages enhanced cell viability and proliferation, accompanied with consistently increased expression levels of proliferative gene (*Ki6*7) and adipose progenitor marker (*Pdgfrα* and *Pdgfrβ*) in preadipocytes (Figure 7B–D). While preadipocytes treated with culture medium from A2B1-knockdown macrophages exhibited the opposite, including decreased cell viability, proliferation and suppressed expression of proliferative gene and progenitor markers (Figure 7E–G). Overall, these data suggested that A2B1 promotes macrophages polarization and proinflammatory cytokines secretion, which may enhance the proliferation of eWAT preadipocytes.

### 3.7. hnRNPA2B1 Controls Proinflammatory Gene mRNA Stability in Macrophages

**Figure 8:**
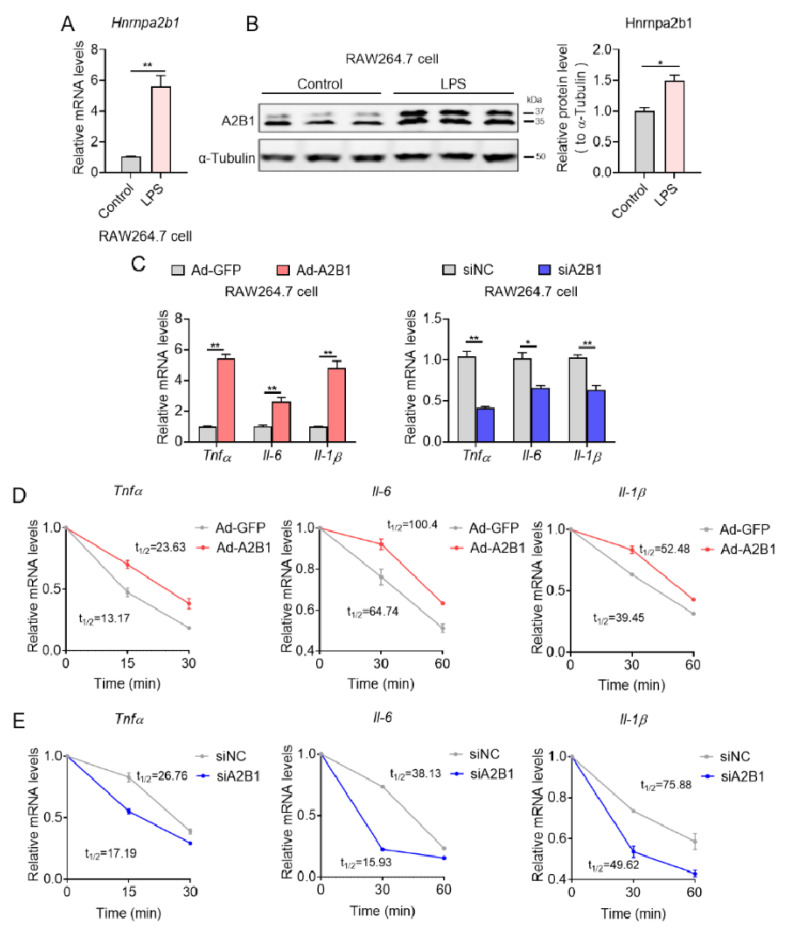
hnRNPA2B1 controls proinflammatory genes mRNA stability in macrophages. (**A**,**B**) hnRNPA2B1 mRNA and protein levels in Control and LPS-treated macrophages. (**C**) Relative proinflammatory genes mRNA levels in GFP and A2B1 overexpression (Ad-A2B1) or in NC and A2B1 knockdown (siA2B1) LPS-treated macrophages. (**D**,**E**) Relative proinflammatory genes mRNA levels in GFP and A2B1 overexpression (Ad-A2B1) or in NC and A2B1 knockdown (siA2B1) LPS-treated macrophages upon transcriptional inhibition with actinomycin D (5 μg/mL) at 0, 15, 30 and 60 min. Results are shown as mean ± SEM; * *p* < 0.05, ** *p* < 0.01.

We then set out to unravel the mechanism of how modulation in A2B1 levels affected inflammation and M1 macrophage polarization. Consistent with in vivo results, we found significant increase in A2B1 mRNA and protein levels in LPS-treated RAW 264.7 macrophages, an in vitro model that favors M1 macrophages polarization, compared to untreated controls (Figure 8A,B). In addition, in LPS-treated M1 macrophages, adenovirus-mediated A2B1 overexpression (Ad-A2B1) further increased relative pro-inflammatory genes mRNA expression levels, while A2B1 knockdown (siA2B1) decreased these proinflammatory genes (Figure 8C). Furthermore, we detected the level of phosphorylated NF-kB to evaluate its activation, and we found that in RAW264.7 macrophages silenced with A2B1, the level of p-NF-kB p65 did not change, indicating that the reduction in pro-inflammatory cytokine production was not due to changed NF-kB signaling (Figure S1). We and others have recently demonstrated that A2B1 is an important posttranscriptional regulator that functions by stabilizing mRNA via its binding to the 3′ untranslated region (3′ UTR) of target mRNA [11,22]. We thus examined whether A2B1 regulated mRNA stability of proinflammatory genes during M1 macrophage polarization. Indeed, we found that in LPS-treated RAW 264.7 macrophages, A2B1 overexpression significantly increased half-life of pro-inflammatory genes mRNA, while A2B1 knockdown resulted in reduced mRNA half-life (Figure 8D,E), suggesting A2B1 regulated M1 macrophages polarization by enhancing the mRNA stability of proinflammatory genes.

## 4. Discussion

Innate immune cell dysfunction, especially impaired macrophage polarization, contributes to the progression of inflammatory diseases, including IBD and obesity [23]. Macrophages can differentiate into two subpopulations: M1 and M2 macrophages. Macrophage polarization and its derived cytokines, including TNFα and a series of interleukins, are associated with the pathogenesis and progression of many inflammatory and autoimmune diseases, such as antimicrobial defense, anti-tumor immune responses, obesity and metabolic diseases [24]. It has recently been shown that posttranscriptional regulations including m6A modification are closely linked to inflammation. For example, the mRNA m6A reader YTHDF2 inhibits pro-inflammatory pathway and maintains hematopoietic stem cell function [25]. Posttranscriptional regulation of *Cebpd* and *Cebpb* is directed by IGF2BP2, which in turn enhances genes expression dependent on these transcription factors and impacts the pathological basis of renal autoimmune inflammation [26]. In the present study, we demonstrate that A2B1, an RNA-binding protein critical for mRNA fate determination, affects M1 macrophage polarization and immune responses in acute and chronic inflammatory diseases, including IBD and obesity, suggesting A2B1 might be a potential target for various inflammatory diseases.

Generally, IBD is a recurrent gastrointestinal inflammatory disease closely related to immune dysfunction [27]. Defective innate immune responses promote the development of IBD in three ways, improper response to benign stimulation, no productive clearance of microorganisms, or failure to switch from pro- to anti-inflammatory response that is usually required to alleviate inflammation [28]. Macrophages are gatekeepers for intestinal immune homeostasis and have been shown to be highly involved in IBD progression. Upon damage, circulating monocytes are attracted to the gut, and subsequently, mature macrophages are differentiated for replenishment of resident macrophages. In the pathogenesis of IBD, the aforementioned differentiation process of monocytes into mature M2 type macrophages is disrupted, and the monocytes adapt to the site of inflammation via CCR2 linked chemokines to differentiate into pro-inflammatory M1 type macrophages [29]. Concomitantly, inflammatory cytokines derived from M1 macrophages can promote Th1 and Th17 immune response and aggravate epithelial injury [30]. As shown in the present study, at the early stage of IBD, haploinsufficiency of A2B1 attenuates M1 macrophage polarization via suppression of proinflammatory gene mRNA stability, indicating that manipulation of RNA-binding protein A2B1 might be a potent strategy to control macrophages polarization, maintain proper responses and relieve IBD. Since A2B1 has been shown to regulate mRNA fate through various mechanisms including A2B1 translocation and mRNA nucleocytoplasmic trafficking, whether A2B1 also modulates key inflammatory messenger RNAs through other mechanisms during the IBD process still need further investigations.

Obesity is defined as excessive fat accumulation in adipose tissue and a state of chronic systemic inflammation, and it features activated adipose tissue macrophages (ATM) as M1 subtypes and increased inflammatory cytokines [31,32]. Interestingly, we have previously shown that A2B1 regulates mRNA stability of key metabolic genes and promotes thermogenic capacity of beige adipose tissue [11]. Considering A2B1′s function in thermogenesis, it is reasonable to predict that A2B1 HET mice would be prone to obesity and metabolic diseases under a high-fat diet. However, we found that compared to WT mice, A2B1 HET HFD mice did not show obvious metabolic dysfunctions such as insulin resistance, while they even had an improved serum lipid profile and a marginal reduction in fat mass, possibly due to the significantly decreased epididymal fat weights, as enlarged eWAT has been revealed to play a detrimental role in metabolic homeostasis [33,34]. Furthermore, HFD-fed A2B1 HET mice showed reduced inflammation and M1 macrophages, which may also make the mice healthier. We further found that the adipocyte sizes of A2B1 HET eWAT were comparable to WT, which may be contributed both by reduced preadipocytes and M1 macrophage numbers in eWAT of A2B1 HET mice, since macrophages has been shown to control preadipocytes proliferation, survival and adipogenic capacity via secreted cytokines [35,36,37]. Of note, since the current study utilized A2B1 HET mice, which affects A2B1 levels on a whole-body basis, the net influence of A2B1 reduction in diet-induced obesity may be due to its regulatory roles in multiple tissues or cell types. Thus, future studies to decipher the complex roles of A2B1 in mature adipocytes, preadipocytes and macrophage via tissue- and cell-specific conditional knockout mice would provide more comprehensive understanding of A2B1 in chronic inflammation, obesity and metabolic diseases.

Taken together, we demonstrated that the RNA binding protein A2B1 regulates DSS-induced colitis and diet-induced obesity by affecting the number of M1 macrophages and proinflammatory gene mRNA stability, suggesting A2B1 may be vital for acute and chronic inflammatory diseases and could serve as a potent therapeutic target.

## Data Availability

No new data were created or analyzed in this study. Data sharing is not applicable to this article.

## Supplementary Materials

The following supporting information can be downloaded at: https://www.mdpi.com/article/10.3390/nu15071555/s1, Figure S1: The NF-kappaB activation is not altered in hnRNPA2B1-silenced macrophages; Table S1: Primers used in qPCR.
