# Supplementary material for: HnRNPA2B1 Aggravates Inflammation by Promoting M1 Macrophage Polarization
Source: Nutrients. 2023 Mar 23;15(7):1555. doi: 10.3390/nu15071555 (PMC10096984; doi:10.3390/nu15071555)
Supplement: Supplementary file 1 [file nutrients-15-01555-s001.zip › nutrients-2298862-supplementary.pdf]

Table S1. Primers used in qPCR.

Gene	Forward	Reverse
β-actin	GGCTGTATTCCCCTCCATCG	CCAGTTGGTAACAATGCCATGT
Hnrnpa2b1	CAGGGTAGTTGAGCCAAAACG	TTCCAGACTGCCTATCGGTAA
Tnfα	CCCTCACACTCAGATCATCTTCT	GCTACGACGTGGGCTACAG
Il-6	GGGACTGATGCTGGTGACAA	TGCCATTGCACAACTCTTTTCT
Il-1β	GAAATGCCACCTTTTGACAGTG	TGGATGCTCTCATCAGGACAG
Pdgfrα	ATGAGAGTGAGATCGAAGGCA	CGGCAAGGTATGATGGCAGAG
Pdgfrβ	CAAGAAGCGGCCATGAATCAG	CGGCCCTAGTGAGTTGTTGT
Ki67	ATCATTGACCGCTCCTTTAGGT	GCTCGCCTTGATGGTTCCT

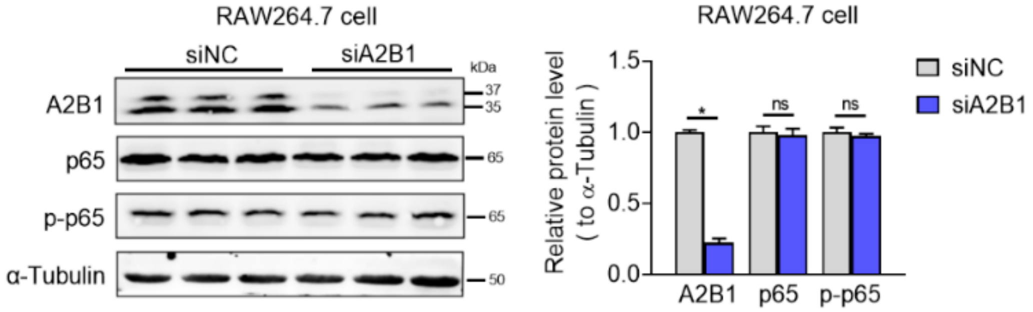

Figure S1. The NF-kappaB activation is not altered in hnRNPA2B1-silenced macrophages. hnRNPA2B1, p65 and p-p65 protein levels in NC and A2B1 knockdown (siA2B1) LPS-treated macrophages. Results are shown as mean ± SEM; ns, not significant; * $p < 0.05$.
